# Innovating through tradition: kava-*talanoa* as a culturally aligned medico-behavioral therapeutic approach to amelioration of PTSD symptoms

**DOI:** 10.3389/fpsyg.2025.1460731

**Published:** 2025-05-27

**Authors:** S. Apo Aporosa, Dennis Itoga, Julia Ioane, Jan Prosser, Sione Vaka, Emily Grout, Martin J. Atkins, Mitchell A. Head, Jonathan D. Baker, Tanecia Blue, David H. Sanday, Mahonri W. Owen, Chris Murray, Karthik Sivanathan, Tua’ipulotu W. Cuthers, Anau Mesui-Henry, Mary-Jane McCarthy, James Bunn, Ifereimi Waqainabete, Helen Turner

**Affiliations:** ^1^Division of Health, University of Waikato, Hamilton, Aotearoa New Zealand; ^2^Hawaii School of Professional Psychology, Chaminade University of Honolulu, Honolulu, HI, United States; ^3^School of Psychology, Massey University, Auckland, Aotearoa New Zealand; ^4^Psychology Worx, Hamilton, Aotearoa New Zealand; ^5^Te Kura Mata-Ao School of Engineering, University of Waikato, Hamilton, Aotearoa New Zealand; ^6^Te Kotahi Research Institute, University of Waikato, Hamilton, Aotearoa New Zealand; ^7^School of Natural Sciences and Mathematics, Chaminade University of Honolulu, Honolulu, HI, United States; ^8^Veterans Affairs Pacific Islands Healthcare System, Honolulu, HI, United States; ^9^Royal New Zealand Police, Wellington, Aotearoa New Zealand; ^10^Te Whatu Ora, Auckland, Aotearoa New Zealand; ^11^Institute of Environmental Science and Research Limited (ESR), Porirua, Aotearoa New Zealand; ^12^Drug Science UK, London, United Kingdom; ^13^School of Medicine, University of Fiji, Suva, Fiji; ^14^UN CIFAL Center, Chaminade University, Honolulu, HI, United States; ^15^Center for Indigenous Innovation and Health Equity, University of Hawai’i, Honolulu, HI, United States

**Keywords:** kava, naturalistic use, PTSD, trauma, cultural-based therapy, psychology, *talanoa*, products containing *Piper methysticum*

## Abstract

Levels of post-traumatic stress disorder (PTSD), trauma-related distress, and subsyndromal PTSD, (here “PTS”) among combat soldiers and first responders are of international concern. In the broader population, a PTS global epidemic is attending trauma associated with the threatscape of the Anthropocene (increased extreme weather events, natural disasters, conflict, rising poverty, emerging infectious disease) as well as the legacy of the COVID-19 pandemic. PTS is also a health economic burden, with costs associated with treatment, long-term morbidity, and increased risk of mortality. In the Pacific region, rising PTS is associated with the existential threat of climate change and the economic and social legacy of colonization. There is an unmet therapeutic need for improved and culturally aligned PTS therapies in the Pacific and beyond. Medical standards of care for anxiety/PTS typically involve psychotropic interventions such as benzodiazepines (BDZ), tricyclic anti-depressants and anti-psychotic medications which have addictive potential, are only effective in the short term, are contraindicated for key populations such as the elderly and have significantly problematic track records in indigenous populations. Moreover, systemic racism both drives PTS in indigenous and other marginalized populations and limits the efficacy in such populations of conventional PTS therapies which are not culturally relevant or informed. Here, we describe the development of a novel, but traditionally grounded, approach to PTSD symptomatology in the context of Pacific populations. This approach has two elements: kava is a culturally significant Pacific drink used traditionally and in cultural practice, as a relaxant, to promote dialog in group settings, to aid in sleep and to manage anxiety. Its anxiolytic and sedative properties may link to the presence of kavalactones which are putative low potency γ-aminobutyric acid (GABA) ligands. *Talanoa* is a dialog practice common to most Pacific cultures. Our core hypothesis is that, combined, kava-*talanoa* will outperform current standards of care in PTSD symptom management as a culturally augmented cognitive-behavioral group therapy intervention. In this paper we review supporting literature, describe kava-*talanoa* pilot study findings and planned clinical trials, discuss important open questions, and present recommendations for broad-based transcultural applicability of this approach to global PTS burdens.

## 1 Introduction

Post-traumatic stress disorder (PTSD) and trauma-related distress (subsyndromal PTSD, S-PTSD) are increasing at concerning rates globally including in Aotearoa New Zealand (ANZ) and the wider Pacific (Bromet et al., 2018:325; Cameron et al., 2019:1). Populations at risk include well-recognized PTSD groups such as military and first responders including police (Thompson, 2014; Wilson et al., 2016; Cameron et al., 2019:1; Brook, 2020; Police News, 2021; Molyneux, 2022; Schwanecke, 2022). In addition, PTSD is becoming endemic in the broader population. Topical Pacific examples of the latter include the mental health effects of COVID, the impacts of climate change and extreme weather events (e.g., Cyclone Gabrielle in ANZ, the Lahaina fires on Maui in the Hawaiian Islands, and displacement of island communities by seawater rise) (Hong et al., 2021; Crimp, 2023; Rush, 2023). Medical standards of care for anxiety/PTSD typically involve drugs such as benzodiazepines, tricyclic anti-depressants and anti-psychotics. These drugs are problematic because they are addictive, efficacious only in the short term (Dodds, 2017), are contraindicated for key populations such as the elderly and have problematic track records in indigenous populations (Dell et al., 2012; Fick et al., 2015). This creates an unmet therapeutic need for efficacious drug regimens that are safer and non-addictive. Similarly, while it is well-understood that drug therapies benefit from augmentation with cognitive-behavioral therapies (CBT) (Hollon et al., 2014; Dunlop, 2016), there remains a need to tailor CBT-type therapeutic approaches to the specific needs of non-Western ethnicities, such as Pacific peoples. This paper describes the potential that kava (from the *Piper methysticum* plant), a clinically-validated anxiolytic (Sarris et al., 2013a,b,^2019^), can be effective in treating PTS when inclusive of CBT augmentation – *talanoa*, a Pacific form of “talk therapy” – in culturally-aligned Pacific kava-use spaces. We present supporting literature, describe pilot data, planned clinical trials and open questions that drive recommendations for the regional and potentially global applicability of this model to help PTSD patients.

## 2 Shared understanding: background on PTS and its multi-component compounding in Pacific peoples

Post-traumatic stress disorder (PTSD) is a psychiatric disorder that can occur in people who have directly experienced a traumatic event, witnessed a traumatic event that impacted another, learned of a violent or accidental event to a close family member, or has been persistently exposed to aversive details of traumatic events. Subsyndromal PTSD includes the same symptoms as PTSD (i.e., disturbed sleep and nightmares, flashbacks, avoidance behavior, hypervigilance, negative self-talk, difficulty with concentration, memory and decision-making and interference with social activities and/or work [Harvey et al., 2015]), although symptoms do not meet the full diagnostic criteria. As such, and of concern, most subsyndromal PTSD goes undiagnosed and therefore untreated, with reasons including trauma-related avoidance behavior (Gershuny and Thayer, 1999; Wilson et al., 2016; Bromet et al., 2018). Avoidance behavior is also a dominant inhibitor to PTS therapy efficacy (Watkins et al., 2018). Untreated, subsyndromal PTSD can develop into PTSD, particularly with exposure to further stressful events (Clayton and Gebbie, 2014; Harvey et al., 2015).

The World Health Organization (WHO) categorizes PTS as a significant burden on populations, healthcare systems and economies (Bromet et al., 2018). Those who experience a traumatic event are at higher risk of developing comorbid physical conditions ranging from chronic pain to gastrointestinal diseases, and vascular and neurological conditions with concomitant negative impacts to career, education, family, and social relationships (Kornfield et al., 2012; Harvey et al., 2015; Wilson et al., 2016; Bromet et al., 2018). Koenen et al. (2017) explain that PTS “often leads to very serious interpersonal and occupational challenges, and has been estimated to result in 3.6 days of lost productivity per month. The disorder has been called a ‘life sentence’ due to its association with increased risk of chronic disease, accelerated aging, and premature mortality.” (p. 3) Levels of PTS among combat soldiers and first responders (police, ambulance and fire personnel) in ANZ are concerning. Notably, almost 80% of Māori military personnel are reported to have PTS (Hurihanganui, 2020). Half of serving police are reported as having significant PTS symptoms (Brook, 2020; Police News, 2021; Molyneux, 2022), with Corrections officers identified as having rates comparable to paramedics (Wilson et al., 2016). Cameron et al. (2019) add, “[w]ithin military populations, PTSD has also been associated with high rates of attrition, absenteeism, occupational disability, impaired social functioning, and reduced health-related quality of life” (p. 1).

Healthcare access and racial inequity compound PTS in Pacific peoples: Health inequities that affect minoritized and marginalized populations compound the effects of trauma, and concomitantly confirm the need for therapeutic approaches that address issues of inequitable access to care, geographic isolation and structural racism (Rowe, 2019; Talamaivao et al., 2020; Braveman et al., 2022; Mullan, 2023). Structural racism has been defined as the ways in which “societies foster mutually reinforcing inequitable systems that reinforce discriminatory beliefs, values, and distribution of resources.” (Bailey et al., 2017:1543) Additionally, a lack of care strategies for PTSD that are culturally-based and that mitigate provider shortages perpetuates the marginalization of minoritized and marginalized populations including Pacific peoples.

Organizations such as the American Psychological Association [American Psychological Association (APA), 2021] have committed to “centering race/ethnicity and racism as the key drivers of health inequities,” and the UN Sustainable Development Goals (SDG) indicator framework interlinks *Good Health and Wellbeing* (SDG3) and *Reducing Inequalities* (SDG10). In a nationwide ANZ study which evaluated “mono-culturalism and institutional racism” (Came et al., 2018:132) in the healthcare sector against standards such as the WHO *Health Equity through Action on the Social Determinants of Health* (Marmot et al., 2008), it was found that although “New Zealand prides itself on being a country of social equity, racial harmony and ‘fair play’ … racism has become normalized … [with] government policies and practice, and government action and inaction, contribut[ing] to health inequities, particularly in relation to indigenous peoples’ health” (Came et al., 2018:132,133,138; Sharples et al., 2018).

Pacific peoples with PTSD also face additional structural barriers to treatment availability due to the dearth of primary care and trauma-informed specialists in remote and urban healthcare and provider deserts (Douthit et al., 2015; Simpson, 2020; Statz and Evers, 2020; Nguyen et al., 2021). The first responder/veteran, indigenous and wider Pacific populations experiencing high levels of PTSD are often socioeconomically marginalized from high quality care (Matheson et al., 2019). Together, increasing rates of PTS (Cameron et al., 2019:1), the significant personal and societal costs associated with treatment and long-term morbidity (Kornfield et al., 2012; Harvey et al., 2015; von der Warth et al., 2020), concerns that many of the current therapeutic approaches have limited efficacy (Thompson, 2014), and the impact of structural racism, have driven our team to consider innovative culturally informed therapeutic approaches (Shelby et al., 2008; Kornfield et al., 2012).

## 3 Gap analysis: shortcomings in current PTS therapy, both in general and specific to Pacific peoples

Medical standards of care for anxiety and trauma related disorders are dominated by two therapeutic approaches. The first is Cognitive Behavioral Therapy (CBT), also known as “talk therapy” (originally called Cognitive Therapy with later evolutions into Cognitive Processing Therapy [CPT] and Rational Emotive Behavior Therapy [REBT]) (Beck, 1976). PTSD is “notoriously difficult to treat” (Fauzia, 2023), with a US Veterans Affairs (USVA) report showing only a 53% efficacy rate for CBT among their clients [United States Department of Veterans Affairs (USDVA), 2023]. The second common therapeutic approach is psychopharmacological intervention using medications such as benzodiazepines (BDZ), tricyclic anti-depressants and anti-psychotics. The USVDA reports drugs as having lower efficacy (42%) compared to CBT (Harik et al., 2016). Additionally, these drug interventions are frequently addictive, have short term efficacy and are contraindicated for some populations, such as the elderly (Cutler and Narang, 1984). BDZ in particular are γ-aminobutyric acid (GABA) receptor ligands which are sleep-inducing, relaxant and anxiolytic. However, in short term use they can cause disinhibition and are addictive over medium- to long-term use with their efficacy rapidly degrading as users age (Cutler and Narang, 1984; Kaplan, 2013; Ogawa et al., 2019). BDZ use is associated with increased risk of suicide due to emotional disinhibition, impulsivity, and unpleasant withdrawal effects (Dodds, 2017) at all age groups, and in the elderly they are associated with cognitive decline (Fick et al., 2015). There are also problematic track records of these medications in indigenous communities linked to issues of dependency (Dell et al., 2012).

Sleep quality is also a serious deficit in current and emerging medication approaches. There is broad agreement following two recent ANZ-based studies that quality sleep is a “protective factor … [leading to] fewer PTS symptoms among military [and police]” (Richardson et al., 2020; Police News, 2021). BDZ use can be associated with decreased sleep quality (de Mendonça et al., 2023) and recently emerging therapies are also potentially problematic. A 2020 ANZ Health Research Council *Psychedelics in Medicine Workshop* addressed the emerging evidence that Class A drugs and hallucinogens (including LSD, ketamine, psilocybin and ayahuasca) can improve PTSD symptomology (Grob and Grigsby, 2021; Kwan, 2023). Alongside safety, regulatory and cultural concerns (Halberstadt et al., 2018; Nuha, 2023), sleep quality impacts of these types of drug are likely to affect efficacy in PTS and we need to consider impacts of these drugs on PTS brains that are already sleep-deprived (Safer, 1970; Thomas et al., 2022). There are also concerns about rapidly expanding Class A use in vulnerable populations and at the public health level, especially via a “soft” route.

Similarly to CBT, the cultural adaptation of medication approaches is also an opportunity and a shortcoming when identifying potentially successful therapies in Pacific and indigenous populations (Pihama and Tuhiwai-Smith, 2023:5). The use of observationally or clinically validated traditional or cultural medications (often phytomedicines) is a reflection of medical autonomy for indigenous peoples (Katz, 2017). There is also evidence that access to cultural medicines decreases access barriers and mitigates health-seeking behavioral reticence associated with Western medicine for indigenous peoples (Li, 2017). There is significant literature on the need to provide empirically-based and culturally responsive mental health treatment initiatives, which could include adapting CBT for indigenous patients (Vaka et al., 2016, 2022a,b; Pearson et al., 2019; Gameon and Skewes, 2020; Mafile’o et al., 2024; Vaka, 2025a,b). While research shows that Pacific peoples in Aotearoa NZ are overrepresented in PTS statistics (Ministry of Health, 2019), there are also findings suggesting that Pacific peoples (including Māori) who are strongly connected to their culture, and those who have rejected Western interventions and instead engaged in traditional psychotherapy, psychotropic interventions and conceptualization of treatment, have an increased resilience to developing PTS (Higate, 2009; Kent et al., 2013:220; Katz, 2017; Pihama and Tuhiwai-Smith, 2023:8).

It is important to demarcate that the rejection of Western treatment by Pacific peoples may be a result of several socio-political factors. Some of these factors include physical and mental health resource disparities located in indigenous communities, reduced access to equitable care, health plan coverage limitations, and underrepresentation of licensed culturally diverse professionals, resulting in a lack of indigenous treatment options. Thus, what may contribute to the overrepresentation of PTS amongst Pacific peoples working in military and law enforcement is fundamental bias and disparity embedded within the “modern” care system, as an evolutionary consequence of Western colonization of Pacific islands.

There is, therefore, a growing international effort to identify new and innovative PTS therapeutic approaches that address unmet medical and cultural needs. Based on the experience of this field and understanding of shortcomings, a *de minimus* combination of a non-psychedelic anxiolytic medication (e.g., a GABA agonist), that promotes the “right kind” of restorative sleep quality (Gottesmann, 2002; Rusch et al., 2015), with some form of CBT “should” work. Moreover, augmenting GABA ligands with talk therapies such as CBT may lower the therapeutic dose of a GABA ligand to achieve the clinical outcome desired. However, there is clearly room to address the shortcomings of both the medication component and the behavioral therapy that are in current clinical use. Addressing these issues would be likely to increase their potential effectiveness in, and adoption by, Pacific peoples.

## 4 The therapeutic potential of kava and *talanoa* for reducing PTS symptomology

### 4.1 Kava as a safe, viable BDZ alternative with demonstrated anxiolytic efficacy

Safer alternatives to BDZ may be GABA ligands that operate at lower receptor potency and are part of the cultural medicinal landscape of Pacific peoples. Kava (*Piper methysticum*) is a shrub that grows widely across tropical Pacific Oceania (Lebot et al., 1997; Aporosa, 2019a). Kava contains a class of probable low-potency GABA ligands known as kavalactones (Davies et al., 1992; Jussofie et al., 1994). Extensive Western research in randomized controlled clinical trials and a variety of interventional studies validates kavalactones as having anxiolytic and soporific value with low side effect profiles and a non-addictive character (Sarris et al., 2013a,b,^2019^). Both the kava plant and drink are culturally significant to the peoples of the Pacific with a safe use history stretching back more than 2,000 years (Singh, 2004; Abbott, 2016; Aporosa, 2019a). Kava in its traditionally mixed beverage form typically contains 20 kavalactones in combination with various other active ingredients, including flavokavains and alkaloids (Lebot and Legendre, 2016; Bian et al., 2020; Cheung et al., 2022). Together with its role in formal ceremony where kava is exchanged and consumed in order to facilitate and solidify *vā*/*veiyaloni* (relational connection) (Fa’avae et al., 2016; Aporosa and Fa’avae, 2021), kava is also used as a relaxant, to promote communication in group settings, to augment sleep and reduce anxiety (Lebot and Cabalion, 1988; Aporosa, 2019b,^2024^). Consumers of kava describe its anxiolytic properties as manifesting “a pleasant, warm, and cheerful, but lazy feeling” (Hocart, 1929:59), relaxant effects that can often be felt for several days (Blades, 2016; Aporosa, 2019b). Users and observers are also clear that even after consuming large volumes of kava, reason and consciousness remain unaffected (Aronson, 2008:183) nor does it induce emotional or aggressive behaviors that can be associated with high alcohol use (Hocart, 1929:59; Lemert, 1967:333; D’Abbs, 1995:169). Aporosa (2022b:2) comments, kava’s “ability to induce a relaxed, yet cogent, state in the drinker means it [kava] is commonly used to facilitate quality discussion and decision-making” (also see Henry and Aporosa, 2021:183).

Cognitive interference is also a concern when considering candidate PTS therapies, although kava’s lack of cognitive interference is demonstrated in a recent ANZ government-funded study (Aporosa et al., 2022a). This first of its kind study used a validated (King et al., 2018) somatosensory neuro-diagnostic tool to assess six specific neurological functions (Focus, Accuracy, Temporal Order Judgement [TOJ], Timing Perception, Plasticity and Fatigue) during, and following, six hours of traditionally influenced kava use with the results compared against a non-kava drinking control and applied to driver safety (Aporosa et al., 2022a). TOJ was the only factor that had a statistically significant level of negative change but low effect size (*p* = 0.007301, BF = 6.193058). TOJ is linked to sequencing, or “how well [the] brain is able to keep track of the order of events” (Pawluk, 2018). Our findings suggest that when consumed at traditional use volumes, kava’s effects are subtle and compromise cognition via a disruption to TOJ only. Moreover, the nature of this impairment is not the same as that caused by alcohol, cannabis, and other recreationally consumed substances (Aporosa, 2022a:81; Aporosa et al., 2022a). This research also corroborates ethnographic observations; that kava does not impair reasoned thinking or inhibit quality discussion and relational connection, these being key factors of naturalistic kava use in communities (Aporosa, 2019b).

Kava is safe but its perception is dogged by outdated information and inaccurate comparisons (Aporosa, 2019b). This has in part been fueled by several Pacific groups conflating kava with the social effects of alcohol (often from a religious standpoint [Aporosa, 2019a]), regardless of the fact that this conflation is unsupported by both the different physiological effects of the two substances and by the stark contrast in their societal impacts (Aporosa, 2019b). Other critiques of kava also derive from inaccurate comparisons and exaggerated health and socio-cultural concerns (Aporosa and Foley, 2020). Misinformation linked to reports of hepatotoxicity emerging from Europe in the 1990s [eight cases in total out of millions of users, and solely associated with chemical extraction methods (Schmidt et al., 2005:182–187; World Health Organization, 2007; Showman et al., 2015)] has also driven a culturally-, economically- and medically-damaging anti-Pacific narrative about the drink (Showman et al., 2015). Adding further confusion is a variety of non-naturalistic kava preparations that are now available. These include extracts and nutraceuticals, and flavored pop-culture drinks containing *Piper methysticum* sold in convenience stores and marketed as “kava” (Aporosa, 2024). Similarly, beverages containing *Piper methysticum*, often resembling alcoholic cocktails yet referred to as kava, are sold in café-type settings called “kava bars.” Those “cocktails” and pop-culture drinks can also include adulterants such as kratom (*Mitragyna speciosa*, a leaf from Southeast Asian with addictive opioid mimicking effects) (Carney, 2024). This gives kava a “euphoric hit” (Kim, 2021) which is counter-typical to the effects of *Piper methysticum* in stand-alone ingestion and therefore antithetical to its cultural roots.

Further complicating kava information accuracy is its use-form in research. Most kava psychopharmacology, including that resulting from clinical trials and reported in peer reviewed outlets, consists “of inaccurate effect descriptors and research associated with tablet-form kava use, with that […] understanding often applied to, or overlaid on, kava users who drink in naturalistic traditionally influenced settings” (Aporosa, 2022a:80). In short, while tablets and extracts containing varying constituents from the *Piper methysticum* plant are vastly different to naturalistic kava, these are what are typically used in clinical trials, with the study findings then routinely, and inappropriately, applied to kava when used traditionally.

The WHO kava risk assessment has played a key role in clarifying “the safety of traditional and recreational [kava] beverage,” stating, “On balance, the weight-of-evidence from both a long history of use of kava beverage and from the more recent research findings indicates that it is possible for kava beverage to be consumed with an acceptably low level of health risk” (Abbott, 2016:26). That report also includes data showing naturalist kava to be “dramatically safer” than Paracetamol (Rasmussen, 2005). Further, that safety level is reflected in ANZ, with kava regulated as “food” under the *Australia New Zealand Food Standards Code* (New Zealand Government, 2015) and recent designation as Generally Regarded As Safe (GRAS) in the state of Hawai’i (Fink, 2024). With many of the concerns surrounding kava now largely resolved and the balance of evidence supporting its safety when prepared in its traditionally influenced aqueous form [Food and Agriculture Organization of the United Nations (FAO), and World Health Organization (WHO), 2023], kava is well placed for use as part of a novel therapeutic approach to PTS.

As we consider kava perceptions as a critical component of building a therapeutic approach to PTS, addressing a definition of kava for these purposes seems appropriate. Some researchers, Pacific cultural experts and practitioners argue that Western commodified *Piper methysticum*-containing nutraceuticals and other products including pop-culture drinks etc., as described above, do not meet the definition of kava that is operant in its originating cultures (Procyk and Lebot, 2013; Blades, 2018; Aporosa et al., 2022b; Tabureguci, 2024). If kava is a cultural keystone species inclusive of practice and relational connection informed by 2000+ years of traditional knowledge (Aporosa, 2019a), once those elements are isolated and used in the absence of that cultural umbrella, we argue it loses its function and designation as kava. Therefore, as we lay groundwork for adoption of culturally contextualized kava as a component of a combination medication/CBT approach to PTS, we in turn assert this naturalistic definition of kava for this purpose: kava is a Pacific cultural term and to use it as a catch all for any product containing *Piper methysticum* is appropriative and misleading.

### 4.2 Form matters: naturalistic kava preparation has likely pharmacological and cultural significance

Products experienced as a nutraceutical are typically pharmacologically manufactured tablets or caplets containing extracted and/or micronized *Piper methysticum* and administrated at a maximum daily dose of 300 mg of kavalactones (Bian et al., 2020). Alternatively, mixtures of isolated kavalactones can also be contained in tablet form (MediHerb, 1994). Naturalistic kava settings differ greatly in both pharmacological dose and cultural form. In contrast, traditionally prepared kava is an aqueous beverage made by straining the crushed roots and basal stump of the *Piper methysticum* plant through water (Aporosa et al., 2020). Pharmacologically, at first glance naturalistic kava drinking seems to result in very high doses. In these spaces, it is common for kava consumers to each consume 3.6 liters of kava beverage, equating to more than 5,000 mg of kavalactones, during an average six-hour session (Aporosa, 2014). While this is 15 times the pharmacologically recommended daily dose in tablet form (Aporosa and Tomlinson, 2014), even at such high consumption volumes, research [e.g., WHO (Abbott, 2016)] reports kava to be safe (Aporosa, 2019b), and high volume traditional kava use is not likely to contribute to vestibular disturbance, increase fall risk (Aporosa et al., 2022b), precipitate hepatotoxicity or negatively impact liver function (Schmidt, 2014; Kuchta et al., 2015). Neither does high naturalistic kava use lead to addiction or cause hallucinogenic effects, marked euphoria or interrupted reasoning (Aporosa, 2019b,2022a; Aporosa et al., 2022a). The underlying reason for this may lie in the inherent poly pharmaceutical nature of traditional kava preparations. In other settings such as the “entourage” effect documented for medicinal marijuana and the Jun (emperor), Chen (minister), Zuo (adjuvant) and Shi (messenger) concept for assembly of traditional Chinese medicine, we see that purification and reduction in complexity of traditional medicines often diminishes efficacy and raises side effect profiles (Jansen et al., 2021). The inclusion, rather than simplification of the extensive plant secondary metabolome, allows for the inclusion of accessory molecules, modulators of side effects and compounds that alter pharmacodynamics such as natural excipients. Naturalistic and traditional extraction methods likely exclude phytochemicals that are only extractable with organic solvents and are not part of the therapeutic mechanism of action for kava (or may be actively harmful). However, these extraction methods are likely inclusive of the wider *Piper methysticum* secondary metabolome (in additional to the kavalactones) that is likely to contribute both to efficacy and side effect limitation. In summary, kava in its traditionally prepared and used state is an attractive prospect for the pharmacological component of an envisioned culturally contextualized combination medication/CBT approach to PTS.

**FIGURE 1:**
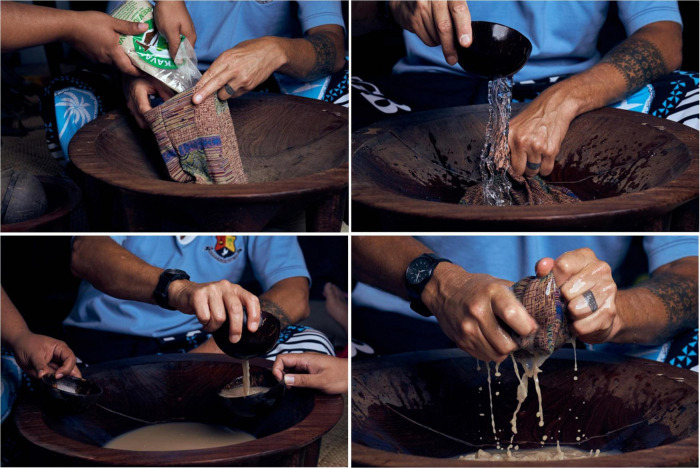
Kava mixing and serving in Tāmaki Makaurau Auckland, Aotearoa New Zealand. Clockwise: 1. dried and pounded kava powder is added to the silk mixing bag; 2. water is added to the *tanoa* or *kumete* (traditional kava bowl); 3. the kava (in the mixing bag) is washed through the water until the desired concentration is achieved; 4. the kava is served in *ipu*/*bilo* (cups made from half-coconut shells). Photographer: Todd M. Henry, 2019.

**FIGURE 2:**
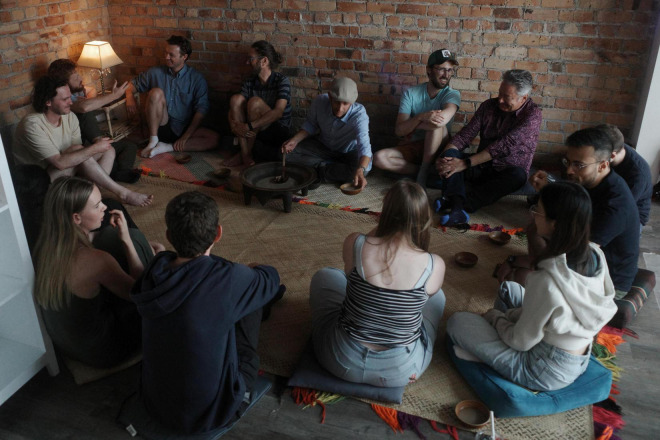
Mixed ethnicity kava users seated on *fala*/*ibe* (woven mats) and engaging in *talanoa* in Tāmaki Makaurau Auckland, Aotearoa New Zealand. Reproduced with permission from photographer: Todd M. Henry, 2022.

The naturalistic model of kava use also has implications for the cognitive-behavioral component of PTS treatment. In naturalistic use settings, which stands in contrast to non-naturalistic products and preparations containing *Piper methysticum* and most commercial spaces called “kava bars,” kava users typically sit on the floor on *fala/ibe* (woven mats that symbolically represent the weaving of *vā/veiyaloni* through respectful *talanoa*) (Faleolo, 2019; Sione, 2023), and drink kava from *ipu/bilo* (cups made from half-coconut shells) served from a *tanoa* or *kumete* [traditional kava bowl; see Figures 1, 2 (following page)] (Christian and Aporosa, 2016). While some of these spaces are relaxed and lack the formalities of ceremony, they are nevertheless underpinned by Pacific respect-based cultural values and protocols, and aimed at cultivating and solidifying *vā/veiyaloni* through *talanoa* (Tago-Elisara, 2020). As mentioned above, fundamental to naturalistic kava use and *vā/veiyaloni* is *talanoa*. *Talanoa* is a form of discussion, a deliberation process encouraging democratic dialog, which underpins gatherings and socialization for Pacific peoples (Robinson and Robinson, 2005:14; Mishra, 2007:45; Aporosa, 2014: 164–165; Fa’avae et al., 2016). The therapeutic “partnership” we propose between kava and *talanoa* is founded on the fact that the pharmacological manifestation of kava is not disruptive of *talanoa* as a stimulant or euphoric would be. Thus, kava is frequently used in culturally informed restorative justice processes and the resolving of intrapersonal and interpersonal conflict (Ratuva, 2002; Cretton, 2005). Moreover, and of particular interest to the present discussion is work by the ANZ Ministry of Health, La Va, Te Pou and independent research that reports *talanoa*’s recognized CBT efficacy among Pacific peoples (Kingi-Uluave and Olo-Whaanga, 2010; Vaka, 2025a,b; Vaka et al., 2016).

### 4.3 Emerging evidence for kava-*talanoa* as an effective intervention for PTS in Pacific peoples

Patient Reported Outcome (PRO) evidence suggests that kava shows considerable potential to improve mental health and to relieve PTS symptomatology. There are several case studies, aggregated for review here, where the combined use of kava and *talanoa* appears to allow PTS sufferers to engage with, and process traumatic events, as opposed to avoiding them. Avoidance is a key barrier to PTS therapy efficacy (Watkins et al., 2018:6) and kava’s anxiolytic effects appear to reduce the triggering of “fear structures” while engaging in processing. Watkins et al. (2018:3) explain that “Fear structures can represent realistic threats, which is normal. However, fear structures can become dysfunctional.” In addition to decreasing avoidant behavior, kava’s long-lasting anxiolytic effects also appear to improve sleep, with quality sleep shown to be a “protective factor … [leading to] fewer PTS symptoms among military [and police]” as reported above (Richardson et al., 2020; Police News, 2021).

Wilson et al. (2018:21) quote a research participant from a semi-structured interview cohort-based study: “[At] our kava session, we talk, and it’s a safe space where you can share things, getting together, we have our therapy session.” Mila-Schaaf and Hudson (2009:118) concur, describing “the kava circle … [as] a space where intercultural negotiation and dialog … [occurs,] responsive to cultural needs of Pacific peoples affected by mental illness.” Tecun et al. (2020:188) cite a recent returnee from combat missions in Afghanistan who commented, “it [kava] helped me deal with my issues … the [psychological] battles that followed after experiencing combat.” Tongan Psychiatrist Dr. Mapa Puloka, who uses kava to facilitate weekly group therapy sessions, reports greater success with clients “just drink[ing] kava and talk[ing]” than he had when using Western psychiatric therapies (Poltorak, 2019). In the field of disaster psychiatry, Weisæth (2020:48) builds on anecdotal reports that culture, and particularly traditional kava use, has the potential “to help people to cope with severe life events.” Weisæth explains that in 1996, Fijian soldiers attached to a United Nations (UN) peacekeeping mission in Lebanon witnessed the aftermath of more than 100 civilians killed by artillery shelling. Those civilians, many of whom were women and children, had sheltered in a church and the Fijian peacekeepers were tasked with the clean-up. Aporosa conversed with two members of that team, who described the scene as an “abattoir” and the psychological impact it had on those tasked with the clean-up. Weisæth was part of a group stress debriefing (GSD) team, sent by the UN to provide trauma intervention support to the Fijian peacekeepers. He noted, “[a]mong their traditional ways of coping with severe stress, the Fiji culture has the ceremonial use of kava drinking … in intense group settings. Observing the obvious value that the interactions in the group had in coming to terms with what had passed made us drop ideas about GSD because we realized that they had their own [i.e., kava-*talanoa*] format for group processing and that a traditional GSD might have disturbed their own way of working through.”

Aporosa has engaged, since 2015, in four visits with UK military personnel recently returned from active service in Afghanistan and, as part of a Fulbright award, with US combat veterans who had retired early due to PTS (Marriner and Aporosa, 2022). He facilitated kava-*talanoa* settings, in which those active and retired military personnel discussed their deployments, combat experiences, readjustment to their environment on return and PTS. Informants subjectively reported the following:

• Reduced rates of PTS symptomology when compared to British/US peers which they attributed to engagement in *talanoa* while consuming kava,
• Combining *talanoa* and kava allowed them to relax while unpacking their combat experiences,
• Kava was also described as improving quality sleep, one informant commented on the “important role kava played in allowing him to sleep without being startled awake,”
• Kava-*talanoa* as a gateway to connect relationally with others who had experienced similar stressful situations and talk about the loss of close friends in a manner that felt peaceful,
• Contrasts between their experience and those of alcohol drinking close peers, for whom alcohol heightened emotions and appeared counterproductive when discussing traumatic events.

The latter point about alcohol is supported by research and further informant testimony. The US Department of Veterans Affairs [United States Department of Veterans Affairs (USDVA), 2022] warns that alcohol use by PTS suffers can exacerbate anxiety and sleep disturbance, entrench avoidance behaviors, and heighten feelings of anger and irritability. UK-based Pacific post-combat soldiers also reported to Aporosa similar concerns about PTS, alcohol, and irritability, with one echoing several others present at the time. He stated that he had stopped consuming alcohol following a combat mission as it tended to heighten tension, which then turned to anger: “I want to rip up everything, I want to smash it, smash my mates, my good mates who are there for me. I can’t stop. But yaqona [kava] is different to beer. You relax, you can talk, it’s ok, you feel better.”

Kava settings with discussions of first-responder stressors and trauma for serving police and Corrections Officers of mixed ethnicities and gender have been facilitated by Aporosa and Sanday in ANZ. Aporosa is a former soldier and policeman (who left the police with diagnosed PTSD), and Sanday is a serving NZ Police Detective Sergeant. In 2021, they collaborated on using the Criminal Investigation Branch (CIB) detective qualifying course as a pilot therapeutic environment for kava-*talanoa*. For the mixed gender, predominantly European participants, it was their first time to sit on woven mats on the floor, drink kava in a culturally influenced setting, and engage in *talanoa*. Following the session, participants provided written feedback that expanded on the value of the experience, its provision of “something I didn’t know I needed at a time I was struggling” and the opportunity for dialog on “our journey in a safe environment.”

## 5 Next steps: necessary clinical trials and open questions

The potential of kava to reduce PTS symptomology has been recognized by the Fulbright Foundation, allowing collaboration between this study and trauma experts and military psychologists in the USA. That work, together with reports from traditionally influenced kava using post-combat personnel in the UK military, have informed the design of clinical trials which are funded by a Health Research Council of New Zealand (HRC) Pacific Projects Award (Marriner and Aporosa, 2023). This trial is a critical step in scientifically establishing the efficacy of kava-*talanoa*, and also aims to address unresolved questions in terms of kava delivery modes. The trial addresses three hypotheses: (1) Kava plus *talanoa* will improve outcomes measured through psychometric assessment and interviews in PTSD and Subsyndromal-PTSD patients, particularly, but not limited to, Pacific persons; (2) That comparison of beverage versus kava caplet delivery methods will identify dose-response relationships and inform design of standardized intervention recommendations; and (3) Interview and patient assessment data will support the soporific/restorative sleep-enhancing effects of kava as a component of its mechanism of action in PTSD and S-PTSD. At the conclusion of this study, we will generate an evidence-based recommended treatment protocol for kava with *talanoa* in the form of a therapeutic practice manual to enable clinical psychologists throughout ANZ (and globally) to safely employ this therapy as part of treatment for PTS.

There are a number of open research questions concerning kava-*talanoa* as a therapeutic option for PTSD and S-PTSD that drive the hypotheses to be addressed in this trial: in traditional settings kava is typically served from a communal bowl and users engage in *talanoa*, which underpins gatherings and socialization for Pacific peoples. Our first question is to ask if the kava-*talanoa* improves PTS outcomes and has particular benefit not only for the 70% of Pacific participants, but also the 30% of Europeans in the study, due to its cultural alignment. Second, in order to eventually design a standardized intervention, we need to know if delivery methods and/or dose standardization are important, which we can address through comparing beverage and caplets which are prepared using traditional aqueous extraction methodology and freeze drying (i.e., a first assessment of caplet delivery that maintains some fidelity to the naturalistic extraction methodology) versus kavalactone caplets. This aim also starts to examine the likely molecular targets of the pharmacological component of kava-*talanoa*, and specifically a controversy in the field as to whether kava ‘*is*’ kavalactones or whether a more complex assemblage of secondary metabolites make naturalistic kava “greater than the sum of its chemical parts.” Also, we acknowledge that caplets could be a necessary component of wider geographical dissemination of this methodology, and thus understanding the efficacy of this delivery modality becomes important. Third, it is postulated that quality sleep is a central component of combatting PTS and we need to assess whether this is a likely mechanistic component of the benefit of kava-*talanoa* and a component of its potential improvement over current approaches to PTS.

Looking forward, this trial will commence mid-2025 and we anticipate the Practice Manual to be available in late 2026. The trial’s lead team is in the process of building an international consortium to examine kava-*talanoa* deployment in Europe and the US in addition to the Pacific. The US arm of the project will be led operationally by the Research Directorate of UN CIFAL Honolulu in collaboration with the Hawaii School of Professional Psychology, both housed at Chaminade University of Honolulu. A National Science Foundation Pacific data science Alliance led by Chaminade will spearhead data analytics and visualization, and the University of Hawai’i Center for Indigenous Innovation and Health Equity (CIIHE) will support outcomes and Practice Manual dissemination in the US-affiliated Pacific. The European includes a team comprising researchers from the British Army, Northumberland University, Worcestershire University, University of South Wales, Veterans National Health Service (NHS) Wales, Drug Science UK, and two third-sector veterans’ charities, Avon and Wiltshire Mental Health Partnership NHS Trust and Woody’s Lodge Veterans Charity. With the goal to recruit non-Pacific peoples in the European arm unlikely to have any frame of reference for traditionally influence kava use, this will provide unique data to triangulate with that of the US and ANZ arms.

Despite the progress made in understanding the potential benefits of naturalistic kava for individuals with PTS, open research questions remain and animate our team for future studies. Addressing these questions in future studies will significantly advance comprehension of kava’s therapeutic potential and its integration into PTS treatment protocols:

Mechanisms of action: What are the precise neurobiological mechanisms through which kava exerts its anxiolytic and sedative effects? Understanding the specific pathways can help tailor treatment strategies to exploit these mechanisms more effectively.

Efficacy in diverse populations and across geographical and cultural distances: How does kava’s efficacy in treating PTS symptoms vary across different populations, including variations by age, gender, ethnicity, and comorbid conditions? What are the barriers and challenges in kava-*talanoa* deployment in different geographical and cultural contexts outside the Pacific? How does the protocol start to vary and how is its efficacy maintained when non-Pacific practitioners are necessarily involved? What training do they need and how would that be best achieved?

Optimal dosage, formulation, and indication: What is the optimal dosage, formulation, and treatment duration of kava for alleviating PTS symptoms without causing adverse effects? Identifying these parameters is essential for maximizing therapeutic benefits while minimizing risks. What subtypes of PTS are suited for treatment with this approach?

Genetic and environmental influencers of response: Are there specific genetic or environmental factors that influence an individual’s response to kava treatment for PTS? Identifying such factors could improve treatment predictability and personalization.

Health economic evaluation: What are the cost-effectiveness and economic impact of incorporating kava into treatment regimens for PTS? Economic analyses can provide insights into the potential for widespread adoption and sustainability of kava-based treatments.

By addressing these open research questions, future studies can elucidate the role of kava-*talanoa* in the treatment of PTS and optimize its therapeutic application, ultimately contributing to better mental health outcomes.

## 6 Conclusion

Kava is a culturally significant Pacific drink used traditionally with cultural practice as a relaxant, to promote dialog in group settings, to aid in sleep and to manage anxiety. Its anxiolytic and sedative properties may link to the presence of kavalactones which are putative low potency GABA ligands. *Talanoa* is a dialog practice common to most Pacific cultures. Our core hypothesis is that, combined, kava-*talanoa* will outperform current standards of care in PTS symptom management as a culturally augmented cognitive-behavioral group therapy intervention. This paper has presented the background, potential mechanisms and evidence base underlying this hypothesis, and compared elements of the kava-*talanoa* approach to current standards of care. We have advanced reasoning for why, both pharmacologically and behaviorally, kava-*talanoa* may specifically benefit Pacific populations for whom is it culturally aligned and where PTS is a health disparity. Kava-*talanoa* has the potential to impact PTS in settings beyond the Pacific, a clear example of how innovation deriving from Pacific indigenous knowledge offers globally applicable solutions.
